# NetCom: A Network-Based Tool for Predicting Metabolic Activities of Microbial Communities Based on Interpretation of Metagenomics Data

**DOI:** 10.3390/microorganisms9091838

**Published:** 2021-08-30

**Authors:** Ofir Tal, Rotem Bartuv, Maria Vetcos, Shlomit Medina, Jiandong Jiang, Shiri Freilich

**Affiliations:** 1 Newe Ya’ar Research Center, Institute of Plant Sciences, The Agricultural Research Organization, Ramat Yishay 30095, Israel; ofirt@volcani.agri.gov.il (O.T.); rotems.email@gmail.com (R.B.); mariavetcos@gmail.com (M.V.); shmedina@volcani.agri.gov.il (S.M.); 2 The Robert H. Smith Institute of Plant Sciences and Genetics in Agriculture, Faculty of Agriculture, The Hebrew University of Jerusalem, Rehovot 7628604, Israel; 3 Department of Microbiology, College of Life Sciences, Nanjing Agricultural University, Nanjing 210095, China; jiang_jjd@njau.edu.cn

**Keywords:** metabolic networks, microbial communities, metagenomics

## Abstract

The study of microbial activity can be viewed as a triangle with three sides: environment (dominant resources in a specific habitat), community (species dictating a repertoire of metabolic conversions) and function (production and/or utilization of resources and compounds). Advances in metagenomics enable a high-resolution description of complex microbial communities in their natural environments and support a systematic study of environment-community-function associations. NetCom is a web-tool for predicting metabolic activities of microbial communities based on network-based interpretation of assembled and annotated metagenomics data. The algorithm takes as an input, lists of differentially abundant enzymatic reactions and generates the following outputs: (i) pathway associations of differently abundant enzymes; (ii) prediction of environmental resources that are unique to each treatment, and their pathway associations; (iii) prediction of compounds that are produced by the microbial community, and pathway association of compounds that are treatment-specific; (iv) network visualization of enzymes, environmental resources and produced compounds, that are treatment specific (2 and 3D). The tool is demonstrated on metagenomic data from rhizosphere and bulk soil samples. By predicting root-specific activities, we illustrate the relevance of our framework for forecasting the impact of soil amendments on the corresponding microbial communities. NetCom is available online.

## 1. Introduction

Organisms take up energy and resources from the environment, convert them into other forms and excrete altered forms back into the environment [1,2,3]. In microbial communities, environmental resources are converted by primary consumers to alternative forms that are accessible to other members of the community [4,5,6,7,8]. Consequently, metabolic activities shape the structure of the community through food chains and trophic interactions. Understanding community-level metabolism is an essential step towards the manipulation and optimization of microbial function. For example, microbial communities in the rhizosphere are shaped by a plant-specific profile of exudates that serve substrate-mediated recruitment of disease-suppressive microbiomes [9,10]. Deciphering the links between resources and community structure can promote the design of amendments that will support a desired function. The perception of ecosystems as a triangle with three sides provides a conceptual framework for the study of microbial activity in soil systems: environment (the dominant resources in a specific sample), community (species dictating a repertoire of metabolic conversions) and function (availability and/or utilization of altered resources). The systematic exploration of these associations becomes possible with the growing number of metagenomics projects and the availability of high-coverage, community-level gene catalogues from diverse ecological samples. Such catalogues not only reveal the dynamics of community shifts but also enable the exploration of their functional outcomes [11]. A considerable effort has been invested in the development of computational approaches for a functional-oriented interpretation of such data and specifically in deciphering the variations in metabolic activity between treatments. In particular, metabolic network approaches provide a framework for translating discrete data from ecological samples into a structured view of biological functions [12,13,14,15,16,17,18,19,20,21,22]. The subsequent conductance of simulations explores associations between the environment and the metabolic potential of the community [23,24,25]. Similar to genomic approaches where species-specific metabolic networks are constructed based on the content of enzyme coding genes [26]; community networks can be constructed based on the functional annotations of metagenomic data [27]. Network-based simulations allow one to address the influence of changing environmental inputs or the functional repertoire of the community (genomic content in the sample) on the network structure and composition.

Here, we report the NetCom tool that was designed to allow the user to apply metabolic-network approaches for exploring the environment-function-structure associations in complex microbial communities, as inferred from metagenomics data. NetCom is based on a previously published framework for the analysis of assembled and functionally annotated metagenomic data [27] that was developed here into a web-tool. This is an addition to the recently published NetMet, a web-tool that applies similar network approaches for the analysis of genomic data of fully sequenced species [26]. NetCom takes as an input a list of enzymatic reactions whose abundance differentiate between two user-defined treatments and generates the following outputs: (i) pathway associations of differently abundant enzymes (ii) prediction of environmental resources that are unique to each treatment and their pathway associations; (iii) prediction of environmental compounds that are produced by the microbial community and pathway association of compounds that are treatment-specific; (iv) network visualization of enzymes, environmental resources and produced compounds that are treatment specific (2 and 3D). Analysis protocol follows the process outlined in [27] and starts with the construction of treatment-specific metabolic networks and prediction of environmental resources that are unique to each treatment. The next step is the use of simulation for the identification of treatment-specific metabolic processes. In contrast with the set of enzyme coding genes-reflecting the full functional potential of species in a sample, actual metabolic performances are environment-dependent and reflect available nutritional sources. The predicted source-metabolites (environmental resources), together with the metabolic potential (the enzymes), allow the independent simulation of metabolic activity in the different treatments [26,28] and explore the specific influence of environmental inputs on metabolic capacities.

The tool is demonstrated on metagenomic data from rhizosphere and bulk soil samples. The rhizosphere is the soil known as the area that is directly under the influence of living roots. The rhizobiome is known to be strongly influenced by plant roots activity. These act as selective nutritional sources for phytochemicals that stimulate and support the enrichment of specific groups of soil microorganisms [29,30,31,32,33,34,35,36,37,38,39]. A published gene catalogue, constructed from genomic DNA that was extracted from the root and respective soil samples of plant crops was used for characterizing a core set of functional genes associated with root colonization [30]. We demonstrate the application of the framework for the analysis of the dataset, as previously published [27], based on its current implementation in NetCom.

NetCom is an easy-to-use tool, designed for the use of non-computational scientists at the aim of allowing researchers to produce predictions based on metagenomic data. Beyond the example case-study presented here, NetCom is a generic tool for the analysis of metagenomic data and was successfully tested on several datasets from various environments. As users’ input contains processed (assembled and annotated) data from a highly diverse community (soil), significant variations in the size of the input files are not expected in datasets from other (e.g., aquatic or host-associated) samples.

NetCom is available online at https://freilich-lab-tools.com/netcom/ (accessed on 22 August 2021).

## 2. Materials and Methods

### 2.1. Description of User’s Input

NetCom receives as an input a single file that contains information on differential abundance of enzymatic reactions in treatments, based on assembled and annotated metagenomic data. Input files are generated by the EdgeR R package [40]. Entities are enzymatic reactions described by EC accessions. EdgeR package classifies the enzymes as associated with Treatment_1, Treatment_2 or not associated. An example input file is provided in File S1, created as previously described in Ofek-Lalzar, Sela, Goldman-Voronov, Green, Hadar and Minz [30].

### 2.2. Description of the NetCom Algorithm

The NetCom algorithm starts with the construction of a meta-network, containing all enzymatic functions included in the user input file. The network is constructed by mapping enzymes to metabolic reactions based on a scheme downloaded from the KEGG database [41] in June 2016, following the procedure outlined in [26,27,42]. Directional edges represent reactions connected by common metabolites (nodes). The set of metabolic reactions and its organization in the metabolic network it forms reflect nutritional dependencies on the environment [43]. Analyzing the topology of the metabolic networks with the graph theory-based strongly connected components (SCC) algorithm is applied to predict the set of metabolites acquired from the environment [43,44,45]. In NetCom, an environmental proxy is generated for three networks: the full meta-network and two sub-networks of differentially abundant reactions. The environmental proxy is a list of metabolites that are predicted to be externally consumed from the environment (‘environmental resources’). Predictions are based on the implantation of Tarjan’s SCC [46] in the NetworkX 2.5 python package. Since the treatment-specific sub-networks were constructed based on differentially abundant enzymes only, they are highly fragmented, leading to a prediction of artificial source-metabolites [27]. Hence, metabolites representing environmental resources that were identified for treatment-specific sub-network, are compared to those identified for the full meta-network. Only metabolites present in both sets are further considered within the environment proxy list (Figure S1).

To predict metabolic activities in each environment we made use of the Expansion algorithm [26,28]. The network expansion algorithm identifies the set of metabolites an organism can synthesize from a given set of precursors. Simulations start with a set of source-metabolites acting as substrates-here the environmental proxy generated by NetCom at the preceding step; it scans the reaction bank for feasible reactions for which all the possible substrates exist; all feasible reactions are then added to the network, their products being the substrates for the next set of reactions. The network stops expanding when no feasible reactions are found. Thus, the full expansion of the network reflects both the reaction repertoire and the primary set of compounds (environmental proxy). Simulations of environmental activity are carried by expanding the full set of reactions-differentially and none-differentially abundant (meta-network), while using treatment-specific sets of environmental resources (that is, the environmental resources predicted by NetCom). Figure S1 illustrates the process of prediction of environmental resources and network expansion.

Enzymes, environmental resources and compounds produced by the expansion process and are treatment specific are mapped to KEGG pathways. Enrichment is determined using the Fisher test requiring *p*-Value ≤ 0.05. Values are adjusted to multiple testing using FDR correction for multiple testing. Visualizations of the metabolic networks produced for each of the treatments by the expansion algorithm are made using Python 3.6 NetworkX 2.5, Plotly 4.14.3 and Matplotlib. Nodes were positioned according to Fruchterman and Reingold algorithm [47]. Nodes with >25 neighbors, typically considered as secondary reactants (H_2_O, etc.), were filtered to reduce the condensed visualization that is typical of highly robust graphs such as those formed by metabolic networks [48].

NetCom code was deposited in https://github.com/ot483/NetCom (accessed on 22 August 2021).

### 2.3. Web Implementation and User Interface

NetCom was implemented in Python 3.6. The web-tool is a CGI built on top of the Dash 1.20.0 package. Following the uploading of the input file information on file content (distribution of enzyme into Treatment_1, Treatment_2 and not_associated categories) is graphically summarized. The user defines the number of minimal and maximal entities in a pathway and pathways to be excluded from the enrichment analysis, the color of enzymes and compounds in the expanded network and the number of network layout iterations.

## 3. Results

We demonstrate here the application of NetCom for the analysis of metagenomic data providing a step-by-step guideline. The data set was created as previously described in Ofek-Lalzar, Sela, Goldman-Voronov, Green, Hadar and Minz [30] In brief, samples for the construction of metagenomic libraries were taken from the rhizosphere (the area under the direct influence of the root) of wheat and the more distant soil not under direct effect, termed here root and soil samples, respectively. The data were sequenced, assembled, annotated and mapped to EC functional identifiers. NetCom is a web implementation of a network-based approach for the analysis of metagenomic data, applied for the analysis of these root-soil metagenomic libraries [27].

### 3.1. Users’ Input

**Figure 1:**
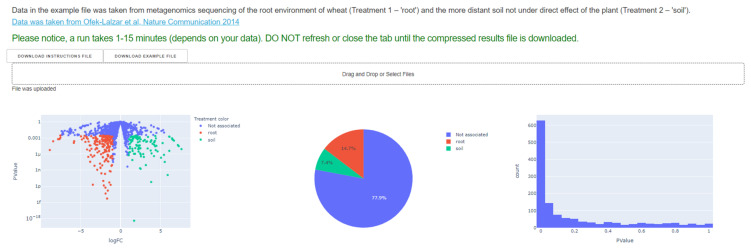
User interface of NetCom following the upload of the input file. Input files are uploaded via the ‘Drag and Drop or Select File’ bottom. An example input file is available (via the ‘Download example file’ grey bottom, top left). Once s valid file is introduced, the three plots on the bottom are generated.

An example input file is provided in the NetCom website (the “DOWNLOAD EXAMPLE FILE”, in Figure 1) and File S1. Valid input files are outputs of the EdgeR R package [40] that uses abundance information of reads associated with accessions (ECs here) across samples from two different treatments and classify each entity as associated with one of the treatments, here root or soil or not-associated. Once a valid input file is introduced, NetCom generates three plots for describing file content while color-stratifying each category (here treatment_1- root, treatment_2- soil, not associated): scatter plot of adjusted *p*-Value vs. logFC, a pie chart showing the fraction of each category and distribution of *p* values (Figure 1).

### 3.2. Differential Abundance Analysis: Characaterization of Differentially Abundant Enzymes and Respective Treatment Specific Environmental Resources and Metabolic Processes

**Figure 2:**
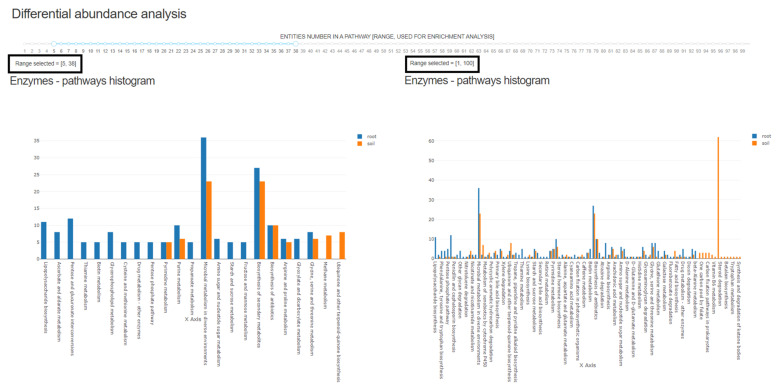
The effect of minimal/maximal pathway size on data presentation. The mapping of differentially abundant enzymes to pathways with (**left**) and without (**right**) filtration of minimal and maximal entities per pathway as selected by the user in the menu shown on top.

Based on the differential abundance association of enzymes, NetCom generates metabolic networks and predicts sets of environmental resources that are characteristics of root vs. soil samples (treatment_1 vs. treatment_2, Figure S1). Lists are provided in the output files root_resources.txt and soil_resources.txt (treatment_1_resources.txt and treatment_2_resources.txt, respectively) that are included in the output directory provided as Data S1. Compounds predicted to provide environmental resources are represented by KEGG Compound accession [49,50,51]. Pathway distribution of both treatment-associated enzymes and environmental resources is presented dynamically in light of the user-defined cutoffs for minimal and maximal pathway sizes (Figure 2). The drop menu allows the user to dynamically exclude pathways, for example, generic pathways such as ‘Metabolic pathways’ or ‘Secondary metabolism’. Mapping of entities into pathways and significance of enrichment in treatment associated entities are provided in the output files root_Enzymes_pathway.csv, soil_Enzymes_pathway.csv (for differentially abundant enzymes in treatment_1 and treatment_2) and root_resources_pathway.csv, soil_resources_pathway.csv (for environmental resources characterized for treatment_1 and treatment_2). Files are included in the output directory provided as Data S1.

In contrast with the set of enzyme coding genes–reflecting the full functional potential of the microbial community in soil, actual metabolic performances are environment-dependent and reflect available nutritional sources. The predicted environmental resources together with the metabolic potential (the enzymatic reactions), allow us to simulate metabolic activity in treatment_1 (root) vs. treatment_2 (soil) environments [26,28] and explore the influence of environmental inputs on metabolic capacities in a given environment. Simulations generate a set of all possible metabolites that can be produced (representing “function”) given (1) a set of feasible reactions identified in the metagenome (representing “community” in the community-function-environment triangle) and (2) sets of compounds representing treatment_1/treatment_2 (root/soil) environments (Figure S1). The resulting networks represent the activity of the community in different samples (root vs. soil) and are composed of shared vs. unique compounds. Lists of compounds that are unique to one of the treatments and their pathway distribution are provided in the output files (root_compounds.txt, root_compounds_pathway.csv, soil_compounds.txt, soil_compounds_pathway.csv included in Data S1).

Most of the enriched pathways were identified in the root environment and include such that were previously reported to be involved in root vs. soil characteristic activities [27]. Some of these root-enriched pathways, including the metabolism of polyketides and anthocyanins are common plant metabolites that are less likely to be abundant with increasing distance from the root [52,53,54,55]. These root unique network functions support the ecological relevance of the expanded environment-specific networks and their relevance for delineating robust versus unique metabolic capacities [27].

### 3.3. Network Parameters

**Figure 3:**
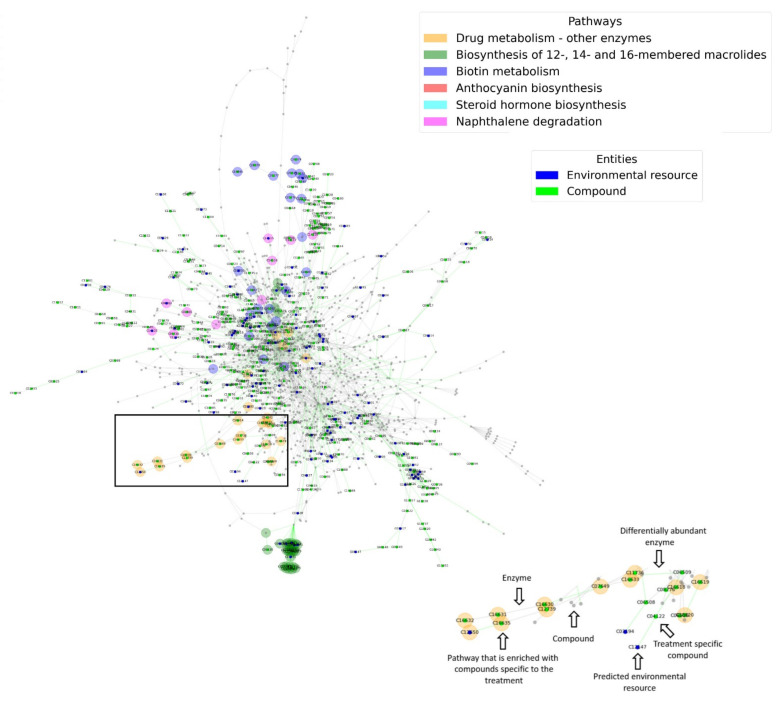
2-D visualization of the metabolic network formed based on metagenomics data. Edges represent enzymes; nodes represent metabolites. Colored edges represent differentially abundant enzymes; Colored edges represent differentially abundant enzymes; colored nodes represent environmental resources and treatment-specific compounds (colors and iterations are selected by the user). Nodes’ background colors (wider circles around the nodes) represent pathways that are enriched (FDR adjusted *p*-Value ≤ 0.05) with network components (nodes) that are unique to the treated samples.

2-D and 3-D visualization of the metabolic networks is produced for each of the treatments. Edges represent enzymes; nodes represent metabolites. A typical dense configuration is characteristics of metabolic networks, reflecting their robustness [48]. Colored edges represent differentially abundant enzymes; light-colored nodes represent the treatment-specific environmental resources predicted based on the topology of the network. The network includes the full set of metabolites (nodes) that the community can synthesize from a given set of precursors (the light-colored nodes), given all feasible reactions (edges); dark-colored nodes represent network components unique to a treatment (here root vs. soil). Color selection and the number of iterations are defined by the user (Figure 3). 2-D Network images and interactive 3-D plots (for the same networks) are included in the output archive (root_network.png, 3D_network_root.html, soil_network.png, 3D_network_soil.html included in Data S1).

Nodes’ background colors (wider circles around the nodes) represent pathways that are enriched (FDR adjusted *p*-Value ≤ 0.05) with network components (nodes) that are unique to the treatment. An interactive dropdown menu allows the user to select enriched pathways that are then presented as 3D dynamic sub-graphs. 3-D plots for enriched pathways are also included in the output files (3D_network_root_5_Naphthalene_degradation.html, 3D_network_root_4_Steroid_hormone_biosynthesis.html, 3D_network_root_3_Anthocyanin_biosynthesis.html, 3D_network_root_2_Biotin_metabolism.html, 3D_network_root_1_Biosynthesis_of_12-,_14-_and_16-membered_macrolides.html and 3D_network_root_0_Drug_metabolism_-_other_enzymes.html in Data S1).

## 4. Discussion

A rapidly growing number of microbial communities are captured by high-coverage metagenomic data as well as complementary ‘omics approaches. The large majority of existing tools and platforms that have been developed for the analysis of this new type of data concern early stages of data interpretation including assembly, gene calling, taxonomic assignment and functional predictions, typically at the single gene level [56]. Network analysis approaches allow contextualization of discrete functions (here metabolic conversions carried by enzymes) and are becoming an essential component in the study of microbial function both at species and community level. Here, we suggest a network-based web-tool for the functional interpretation of metagenomic data. The NetCom tool relies on the description of an enzymatic set identified in assembled and annotated metagenomics data. Given user’s input, this web-tool provides a user-friendly platform for easily producing predictions for treatment-specific metabolic fingerprint through simulating activity in natural-like environments. The predicted source-metabolites (environmental inputs), together with the metabolic potential (the set of enzymes), enable the user to generate a set of all possible metabolites that can be produced (representing “function”) in a given environment by a given community and hence reflect the common notion that metabolism is dynamic and can vary with the addition or depletion of nutrients [57,58]. The environmental approximations are automatically generated by NetCom through the implementation of a computational framework for inferring the set of compounds that organisms consume from their surroundings. This computationally derived set was shown to accurately describe the effective biochemical environments of microbial species, providing a proxy for their natural habitats [59]. In light of the rapid advance of metabolomic technologies, future versions of NetCom can be designed to allow the integration of users’ defined environments as inferred from ‘omics studies.

We demonstrate the application of the tool for the analysis of a metagenomics-derived gene catalogue from the complex microbial communities of plant roots [30]. Our framework was applied for tackling the intricate associations between community structure, community function and metabolic inputs in this important ecosystem [27]. The simulated observations are consitsent common ecological and network concepts. The communal networks are highly robust where the large majority of basic metabolic functions are conserved between environments [48]. Many of these functions that are unique to the root-like environment (vs. soil) reflect the effect of plant exudates and are in agreement with reported observations. Overall, the presented approach was successful in predicting root-specific effects that link the utilization of specific environmental nutrients (here, plant exudates) with community-level activity, pointing at the impact of specific compounds as determinants of the microbial community structure. Notably, the rhizobiome is a central determinant of crop health and yield, hence understanding how to manipulate rhizobium communities towards a desired function is a major agricultural concern [9]. Model-derived interpretation of metagenomic data should serve the formulation of testable predictions. Data interpretation allows researchers to delineate biological signals from complex data and to rationally design possible manipulation strategies that will induce optimized function. Predictions-based design of agricultural practice can include the characterization of the effect of the introduction of environmental treatments to crop fields (that is, adding/depleting specific compounds) [9]. In the absence of appropriate analysis tools and considering the volume of data produced in metagenomics studies, identification of meaningful associations resembles finding a needle in a haystack. Hence, the interpretation platform as suggested here can serve as a starting point for generating experimentally testable hypotheses [60]. Even though demonstrated here on a dataset from arable soil samples, NetCom was designed as a generic tool for the analysis of metagenomic data constructed for microbial communities.

## Data Availability

NetCom code was deposited in https://github.com/ot483/NetCom.

## Supplementary Materials

The following are available online at https://www.mdpi.com/article/10.3390/microorganisms9091838/s1, File S1: An example input File, Figure S1: A schematic illustration of the NetCom algorithm, Data S1: An example output file generated by NetCom using File S1 as user’s input. The file was taken from Ofek–Lalzar, Sela, Goldman–Voronov, Green, HadarandMinz [30] The Following user-defined parameters were applied: treatment-root, control-soil, entities [5,38], hubness-100, network layout iterations-170.
