# Supplementary material for: NetCom: A Network-Based Tool for Predicting Metabolic Activities of Microbial Communities Based on Interpretation of Metagenomics Data
Source: Microorganisms. 2021 Aug 30;9(9):1838. doi: 10.3390/microorganisms9091838 (PMC8468097; doi:10.3390/microorganisms9091838)
Supplement: Supplementary file 1 [file microorganisms-09-01838-s001.zip › FIGURE S1.pdf]

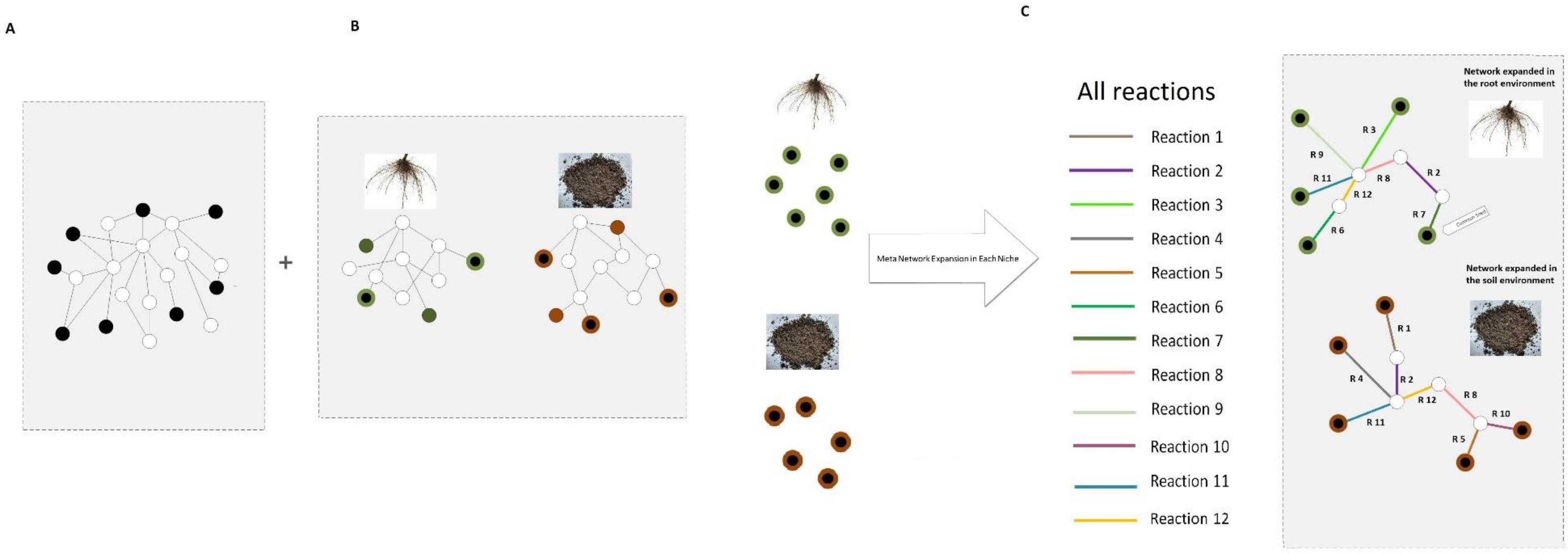

Figure S1 I Schematic illustration of the process of source metabolite selection and network reconstruction. The networks are comprised from metabolites denoted by circles that are connected by reactions (edges). A metabolite that is considered as an environmental resource is colored black. (**A**) Identification of source-metabolites in the meta-network, containing all cross-sample reactions. (**B**) Network reconstruction and prediction of environmental resources for two networks describing the root and soil environments. Each network contains all the differentially abundant enzymes in the corresponding environment. Common resources between a specific environments and the initial meta-network are denoted black with a colored rim. Green shades represent root environment, brown shades represent soil environments. Colored circles (without inner black "pupil") are source-metabolites identified only for the network of differentially abundant enzymes and not for the meta-network and are likely to represent biases formed from the gapped nature of a network which relies

solely on differentially abundant enzymes. (**C**) The common environmental resources between the meta-network and each environment represents the predicted specific environment and were further used for network expansion. The algorithm starts with a set of source-metabolites acting as substrates – here the environmental proxy generated at stage B; it scans the reaction bank for feasible reactions for which all the possible substrates exist; all feasible reactions are added to the network, their products being the substrates for the next set of reactions. The network stops expanding when no feasible reactions are found. Thus, the full expansion of the network reflects both the reaction repertoire and the primary set of compounds (environmental proxy).
