# Supplementary figures and images for: NetCom: A Network-Based Tool for Predicting Metabolic Activities of Microbial Communities Based on Interpretation of Metagenomics Data
Source: Microorganisms. 2021 Aug 30;9(9):1838. doi: 10.3390/microorganisms9091838 (PMC8468097; doi:10.3390/microorganisms9091838)

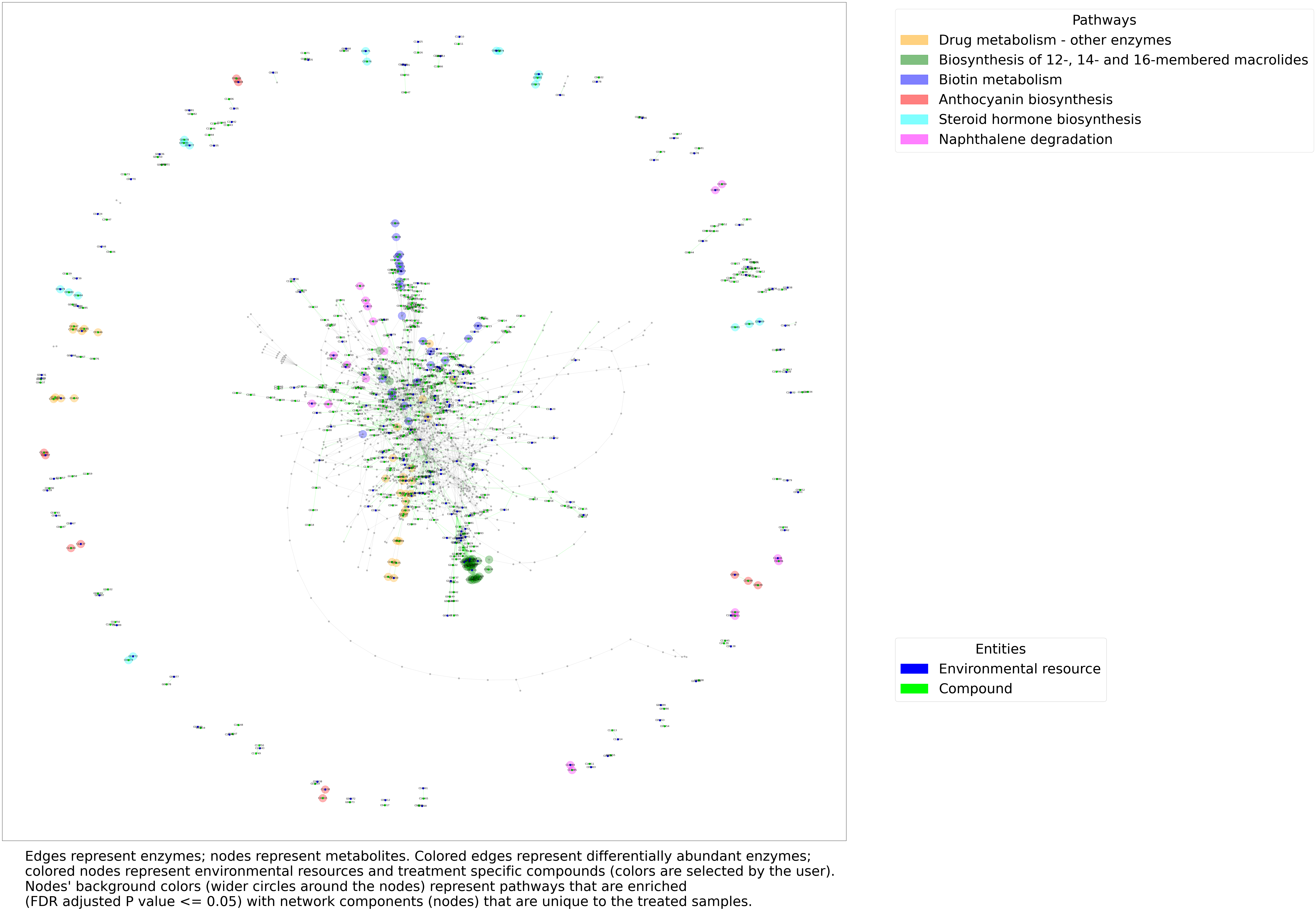

Supplement: Supplementary file 1 [file microorganisms-09-01838-s001.zip › Data S1/root_Network.png]

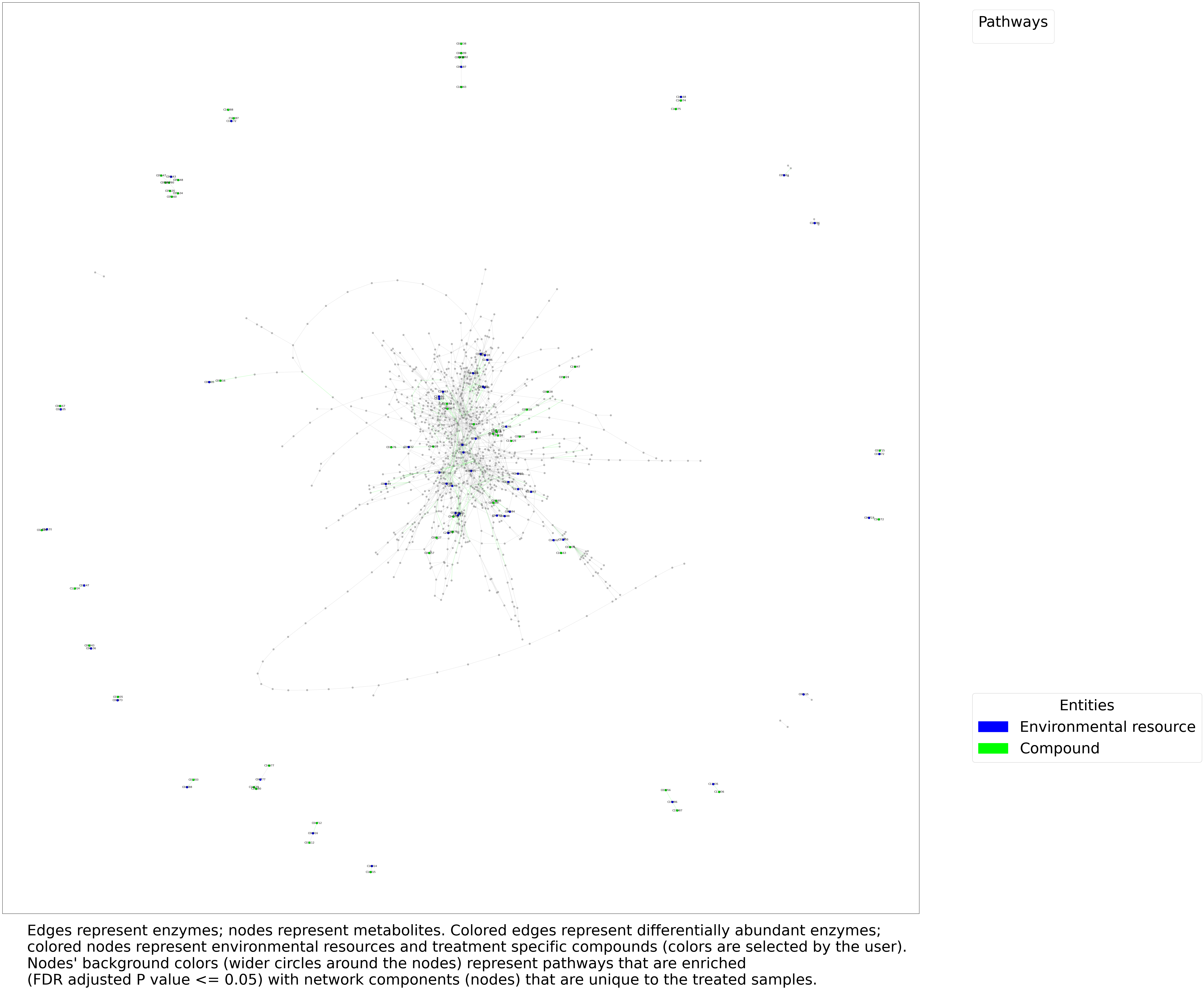

Supplement: Supplementary file 1 [file microorganisms-09-01838-s001.zip › Data S1/soil_Network.png]
